# Supplementary figures and images for: Quantitative environmental DNA metabarcoding shows high potential as a novel approach to quantitatively assess fish community
Source: Sci Rep. 2022 Dec 13;12:21524. doi: 10.1038/s41598-022-25274-3 (PMC9747787; doi:10.1038/s41598-022-25274-3)

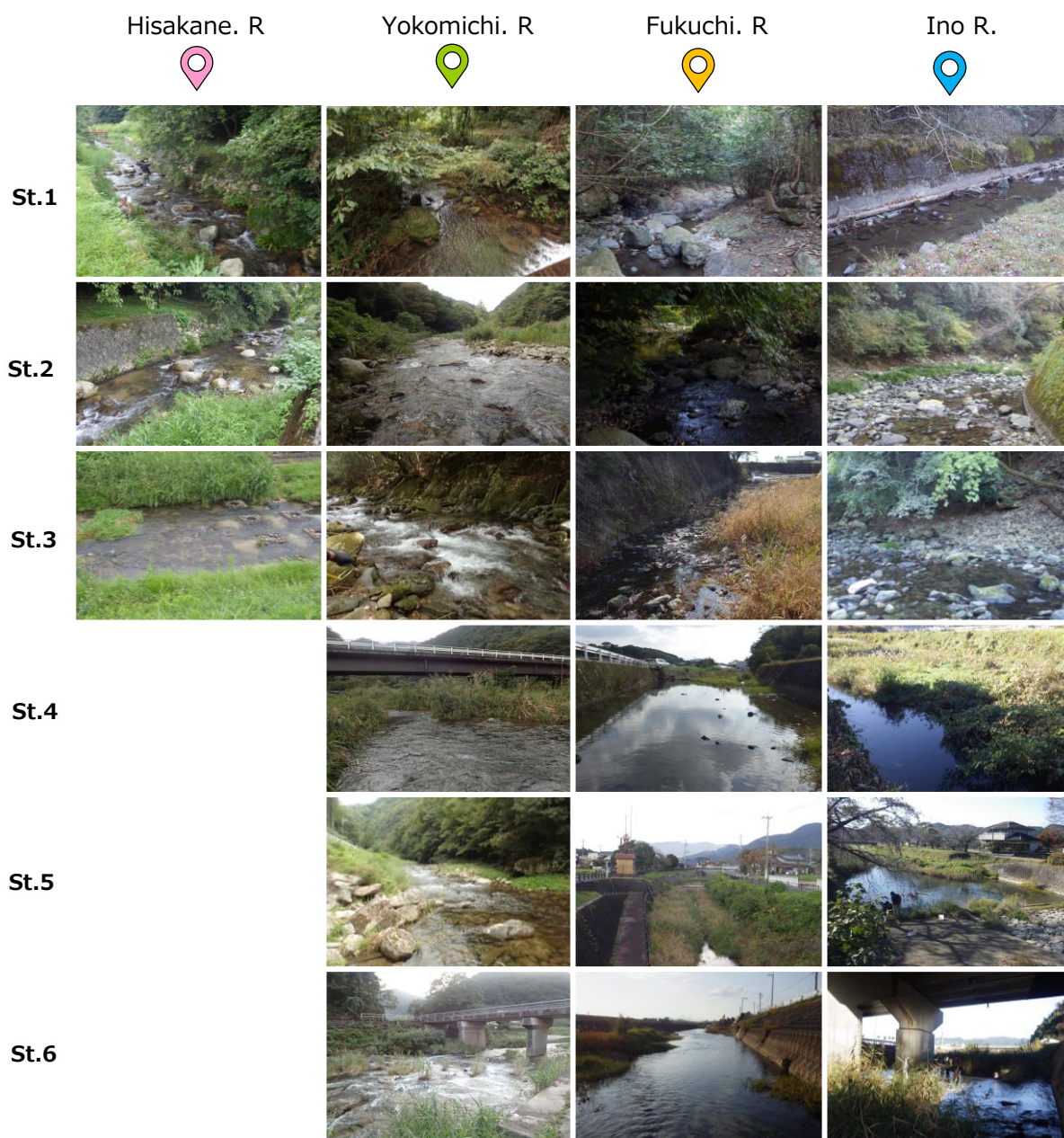

Fig. S1

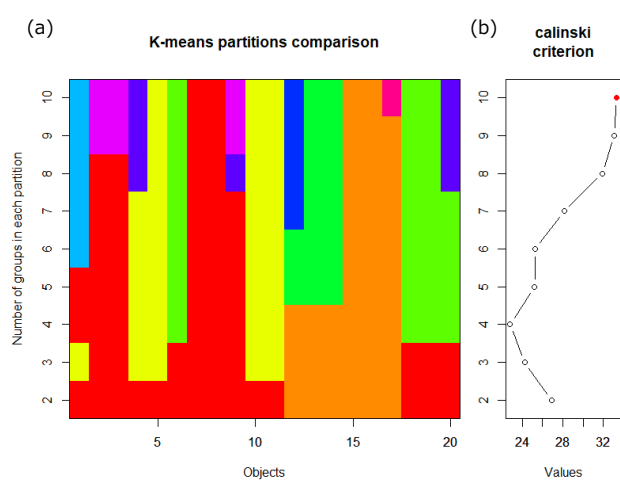

Fig. S2

Supplement: Supplementary file 2 — Supplementary Information 2. [file 41598_2022_25274_MOESM2_ESM.pdf]
