# Supplementary material for: Quantitative environmental DNA metabarcoding shows high potential as a novel approach to quantitatively assess fish community
Source: Sci Rep. 2022 Dec 13;12:21524. doi: 10.1038/s41598-022-25274-3 (PMC9747787; doi:10.1038/s41598-022-25274-3)
Supplement: Supplementary file 3 — Supplementary Information 3. [file 41598_2022_25274_MOESM3_ESM.docx]

**Supplementary figure legends**

Figure S1 Picture of each survey site.

Figure S2 (a) K-means partitions comparison and (b) Calinski values.
